# Use of cesium chloride density gradient ultracentrifugation for the purification and characterization of recombinant adeno-associated virus

**DOI:** 10.1007/s00249-025-01751-1

**Published:** 2025-05-19

**Authors:** Kiichi Hirohata, Shinichiro Kino, Takuya Yamane, Karin Bandoh, Takeshi Bamba, Shawn M. Sternisha, Tetsuo Torisu, Mitsuko Fukuhara, Yuki Yamaguchi, Susumu Uchiyama

**Affiliations:** 1 Department of Biotechnology, Graduate School of Engineering, Osaka University, 2-1 Yamadaoka, Suita, Osaka 565-0871 Japan; 2 Institute of Metabolomics, BYU-Analytica Inc., Suita, Osaka 565-0871 Japan; 3 Division of Metabolomics/Mass Spectrometry Center, Medical Research Center for High Depth Omics, Medical Institute of Bioregulation, Kyushu University, Fukuoka, Fukuoka, 812-8582 Japan; 4 Beckman Coulter Life Sciences, Indianapolis, IN USA; 5 U-Medico Inc., 2-1 Yamadaoka, Suita, Osaka 565-0871 Japan

**Keywords:** Ultracentrifugation, Adeno-associated virus, Viral protein stoichiometry, Vertical rotor, Cesium chloride, Transduction efficiency

## Abstract

Recombinant adeno-associated virus (rAAV) has been widely used as an effective delivery tool in gene therapy. One of the challenges facing the production of high-quality rAAV is optimization of the production and purification methodologies. Cesium chloride density gradient ultracentrifugation (CsCl-DGUC) has been traditionally utilized for rAAV purification; however, few studies have focused on CsCl-DGUC for rAAV purification despite this technique having a great potential for clinical-grade or large-scale rAAV production. In this study, we aimed to explore the unaddressed challenges associated with rAAV purification using CsCl-DGUC. We clarified that AAV capsids assembled by the different stoichiometries of three viral proteins (VP1, VP2, and VP3) showed heterogeneous population in the CsCl density gradient and encapsidated DNA increased the buoyant density differences of these capsids, resulting in the wider distribution. We implemented CsCl-DGUC using a vertical rotor which improved throughput and enhanced the separation of desired AAV particles from impurities. Furthermore, we examined the effect of CsCl exposure during purification, and the presence of residual CsCl in the purified rAAV. This study provides valuable insights into the application of CsCl-DGUC in the manufacturing of rAAV while ensuring adequate efficacy and safety for gene therapy.

**Supplementary Information:**

The online version contains supplementary material available at 10.1007/s00249-025-01751-1.

## Introduction

Advances in genetic engineering techniques have led to the emergence of gene therapy, a promising technique to treat various diseases. In fact, a number of clinical trials are ongoing for adeno-associated virus (AAV)-based gene therapy, resulting in new recombinant AAV (rAAV) drug products appearing on the market for monogenic rare diseases (Mendell et al. 2021). Wild-type AAV (wtAAV) is a non-enveloped virus encapsidates single-stranded DNA (ssDNA) flanked by inverted terminal repeats (ITRs). The icosahedral capsid of wtAAV is composed of 60-mer subunits assembled by three viral proteins (VPs), VP1, VP2, and VP3 at a ratio of 1:1:10. rAAV, designed for gene therapy, has an identical capsid structure to wtAAV, but the rAAV genome contains therapeutic transgenes within its packaging capacity (~ 4.7 kb) instead of wtAAV protein-coding genes. At present, rAAV production is mainly conducted using the baculovirus expression vector system (BEVS) or HEK293 production system. A recent study identified a highly heterogeneous population of AAV capsids with varying VP stoichiometries (Wörner et al. 2021). Furthermore, a positive correlation between the stoichiometric ratio of VP1 and the transduction efficiency in vitro and in vivo for rAAV produced using BEVS has been reported (Wang et al. 2017; Bosma et al. 2018). rAAV produced by HEK293 cells also showed a correlation between VP stoichiometry and transduction efficiency (Onishi et al. 2023).

The current rAAV production system using suspension HEK293 with triple transfection methods could be scaled-up to hundreds of liters in culture volume, whereas such system usually produces more than 50% of AAV particles without a genome (empty particles, EPs), and these EPs in rAAV drug product should be reduced to mitigate the risk of adverse effect. Therefore, it is crucial to eliminate impurities including EPs from the desired AAV particles with a full-length genome (full particles, FPs) to ensure the efficacy and safety of rAAV drug products (Wright et al. 2014). As a result, there are demands for the development of purification processes that can handle large volume of unpurified rAAV. Among the downstream purification methods, cesium chloride density gradient ultracentrifugation (CsCl-DGUC) is attracting renewed attention to prepare highly purified FPs. Although the similarity in physicochemical properties between FPs and EPs is a major barrier for rAAV purification, CsCl-DGUC can achieve high-resolution separation based on buoyant density differences. In addition to the separation of FPs and EPs, it was noteworthy that FPs formed two bands with different buoyant densities (Wang et al. 2016). Recently, it was found that these FPs with different buoyant densities were composed of capsids with distinct VP stoichiometries; FPs with low buoyant density had a higher stoichiometric ratio of VP1 and VP2 than FPs with high buoyant density (Onishi et al. 2023; Hirohata et al. 2024). Moreover, density gradient equilibrium analytical ultracentrifugation (DGE-AUC) enabled quantitative analysis of the heterogeneous FP population (Hirohata et al. 2024). Therefore, CsCl-DGUC has tremendous potential to recover high-activity FPs. However, the theoretical explanation is lacking for the separation of FPs with different VP stoichiometries in the CsCl density gradient. EPs should also exhibit two or multiple bands caused by the capsids with different VP stoichiometries, but unlike FPs, this has not been observed. A comprehensive understanding of the separation capabilities of CsCl-DGUC is essential for improving rAAV manufacturing and developing safer, more efficacious therapies.

Furthermore, the challenges of conventional CsCl-DGUC using swinging-bucket or fixed-angle rotors are time-consuming to complete and also less scalable compared with chromatography-based purification processes (Tomono et al. 2016). Thus, development of CsCl-DGUC using different rotor types has been an area of interest to overcome these limitations. In fact, it was reported that CsCl-DGUC using a zonal rotor effectively separated FPs and EPs on large-scale over a short time period (Wada et al. 2023). Vertical rotors have also attracted attention for ultracentrifugation-based purification process of viral vectors since vertical rotors have the advantage of short path lengths which lead to shorter centrifugation run times for sufficient separation. Nevertheless, there are few studies demonstrating the application of vertical rotors to rAAV purification. Another concern was proposed that long-term exposure to CsCl lowers the transduction efficiency of rAAV (Auricchio et al. 2001), despite CsCl-DGUC purification being used for the manufacturing of clinical-grade rAAV (Clément et al. 2016). CsCl intake has been considered as a potential cancer treatment although high levels have been linked with health risks (FDA 2018). Hence, CsCl should be removed by a dialysis step or ultrafiltration and diafiltration step after CsCl-DGUC in rAAV manufacturing; however, experimental data are lacking on the effect of CsCl on the transduction efficiency of rAAV.

In this study, we examined the separation of AAV particles with different VP stoichiometries by simulations and DGE-AUC experiments. As a result, we clarified that both EPs and FPs with expected different VP stoichiometries exhibit heterogeneous population in the CsCl density gradient and encapsidated DNA increased the buoyant density differences between these capsids, leads to wider distribution for FPs. Next, we investigated the separating power of CsCl-DGUC using a vertical rotor. A swinging-bucket rotor has been routinely used for CsCl-DGUC purification; however, a vertical rotor has the potential to achieve better separation of FPs and EPs more rapidly. Finally, the transduction efficiency of rAAV using HeLa cells was investigated which enabled assessment of the CsCl-DGUC purification process. We evaluated the in vitro efficacy of rAAV in the situation of undergoing CsCl-DGUC and discussed the relationship between residual CsCl or CsCl exposure and the transduction efficiency of rAAV.

## Results and discussion

### Separation of FPs and EPs with different VP stoichiometries in the CsCl density gradient

**Fig. 1:**
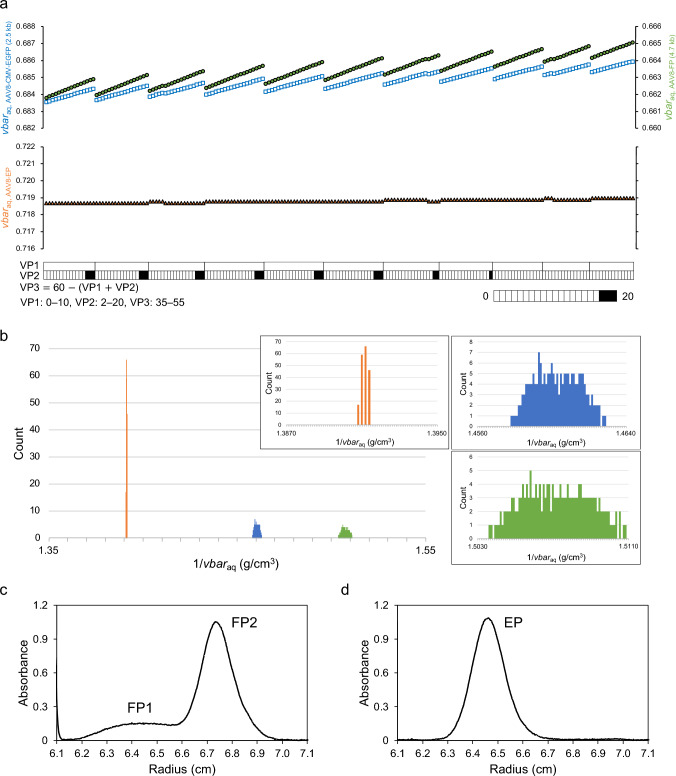
For AAV8-EP (orange), AAV8-CMV-EGFP (blue), and AAV8-FP (green) with different VP stoichiometries, **a** *vbar*_aq_ variations; Gray scale bar represents the stoichiometric ratio of VP1 or VP2 in 60-mer subunits and starting from the left, the plots correspond to the numbered capsids in the list (Supplementary Information), and **b** 1/*vbar*_aq_ distribution; The scale of 1/*vbar*_aq_ values in the overall graph is in 10^–2^ increments, and those in the enlarged graphs are in 10^–4^ increments, all in the same 1/*vbar*_aq_ variation range. DGE-AUC equilibrium profiles of **c** AAV8-CMV-EGFP under the conditions of 2.90 M (1.370 g/cm^3^) CsCl and 30,000 rpm and **d** AAV8-EP under the condition of 2.50 M (1.320 g/cm^3^) CsCl and 28,000 rpm. FP1 and FP2 are two populations of AAV8-CMV-EGFP with low and high buoyant densities, respectively. Both results were detected at a UV wavelength of 230 nm

First, we constructed a matrix of the theoretical values of molecular weight (Mw) and partial-specific volume in aqueous solution (*vbar*_aq_) for FP and EP with different VP stoichiometries. Two FPs were considered, AAV8-CMV-EGFP with a DNA length of 2.5 kb and AAV8-FP with a DNA length of 4.7 kb (GC content of 50%). The Mw and *vbar*_aq_ values were obtained for a total of 188 capsids with a range of stoichiometric ratios of 0–10 for VP1, 2–20 for VP2, and 35-–55 for VP3, which includes the majority of capsids based on previous findings (Wörner et al. 2021). The calculation procedure is described in the Materials and Methods section, and a summarized list is provided in the Supplementary Information. Plots of the *vbar*_aq_ values for AAV8-CMV-EGFP and AAV8-FP, and AAV8-EP with different VP stoichiometries are shown in Fig. 1a. The variations in the *vbar*_aq_ values were larger in the order of AAV8-FP, AAV8-CMV-EGFP, and AAV8-EP. Moreover, all of them included capsids that have almost identical *vbar*_aq_ values with distinct VP stoichiometries.

Although the *vbar*_aq_ value of AAV particle can be determined from amino acid and DNA compositions, it should be noted that the *vbar* under actual solvent conditions differs depending on the solvent composition such as the Na^+^, Cs^+^, PO_3_^˗^, and Cl^˗^ concentrations, as a result of the hydration effect on the particle. Thus, the buoyant density of AAV particle should be the reciprocal of the total value of *vbar*_aq_ and a constant that reflects the hydration effect in the CsCl density gradient. Here, assuming that the constant remains relatively unchanged when VP stoichiometry varies and the same DNA is encapsidated in the capsid, the reciprocal of the *vbar*_aq_ (= 1/*vbar*_aq_) population reflects the experimental buoyant density population in the CsCl density gradient for both FP and EP.

Based on the above discussion, the 1/*vbar*_aq_ values were determined in 10^–4^ increments for 188 capsids of AAV8-FP, AAV8-CMV-EGFP, and AAV8-EP as shown in Fig. 1b. All three AAV8 were distributed in an uneven Gaussian shape as expected from the *vbar*_aq_ variations, AAV8-EP was distributed across a narrow 1/*vbar*_aq_ range, whereas AAV8-CMV-EGFP and AAV8-FP were distributed across wider ranges. Therefore, it is indicated that EP has a single peak while FP has a broader peak in the CsCl density gradient even in the case of exactly the same heterogeneity of VP stoichiometries of AAV capsids. In addition, this uneven peak widened by the presence of DNA encapsidated in FP may contribute to the observation of two or multiple apparent peaks.

To confirm the above simulation results, we performed DGE-AUC experiments to obtain the profile under different experimental conditions. In DGE-AUC, a relatively high rotor speed (42,000 rpm) was selected to determine the FP/EP ratio for purity assessment within the short path length of an AUC cell (Hirohata et al. 2024). DGE-AUC equilibrium profiles for AAV8-CMV-EGFP exhibited two main peaks (FP1 and FP2) with the same ratio of UV absorbance at 260 nm and 280 nm (A260/A280) (Fig. S1a). It must be recognized that if there is heterogeneity of encapsidated DNA, the observed peak gets broader or additional peak is observed with higher buoyant density as A260/A280 increases. AAV8-EP exhibited a single peak at a lower radial point, corresponding with the decreased buoyant density of AAV8-EP (Fig. S1b). Here, the lower the rotor speed, the shallower the density gradient resulting in broader peak, whereas the higher the rotor speed, the steeper the gradient resulting in sharper peak (Sternisha et al. 2023). We next optimized the rotor speed and initial CsCl concentration for the observed peaks. Figure 1c shows the DGE-AUC equilibrium profile of AAV8-CMV-EGFP at 30,000 rpm starting from 2.90 M CsCl. Similarly, the DGE-AUC equilibrium profile of AAV8-EP at 28,000 rpm with an initial concentration of 2.50 M CsCl is shown in Fig. 1d. The observed peaks were broadened in both profiles because of the shallow CsCl density gradient; however, the same populations were observed under different conditions. Thus, we concluded that the observed profile exactly reflects the heterogeneity of VP stoichiometries in the capsid especially for FPs. We note that the heterogeneity of VP stoichiometries in the assembled capsids, in practice, depends on the production systems or plasmid constructs; however, CsCl-DGUC and DGE-AUC are effective for evaluating the heterogeneity of VP stoichiometries in FPs.

**Fig. 2:**
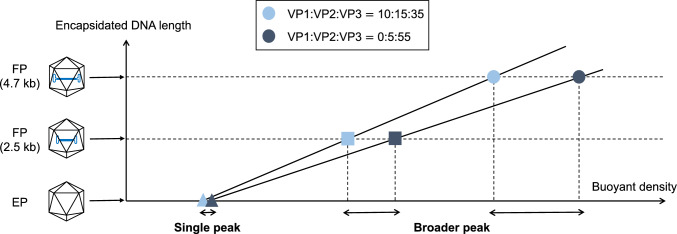
Scheme of buoyant density distribution of capsids with different VP stoichiometries in the CsCl density gradient. Plots of sky blue and dark blue represent AAV capsids with different VP stoichiometries (VP1:VP2:VP3 of 10:15:35 and 0:5:55, respectively). Triangle, square, and circle plots correspond to capsids with DNA lengths of 0, 2.5, and 4.7 kb, respectively

Correspondence between the simulation and experimental results was demonstrated, we have reached a systematic understanding. As shown in Fig. 1a, it was determined that *vbar*_aq_ variations of AAV8-FP with a DNA length of 4.7 kb were amplified compared with AAV8-CMV-EGFP (DNA length of 2.5 kb), and this result is clearly independent of AAV serotypes. Moreover, AAV8-EP had a much smaller *vbar*_aq_ variation than either those of AAV8-EGFP or AAV8-FP. In summary, the existence of encapsidated DNA enhances the buoyant density variations of capsids with different VP stoichiometries, and the variation depends on the length of the encapsidated DNA as shown in Fig. 2. As a result, FPs with different VP stoichiometries can be separated and fractionated under optimized conditions, allowing for the recovery and enrichment of FPs with higher biological activity.

### Investigation of CsCl-DGUC for rAAV purification using a vertical rotor

**Fig. 3:**
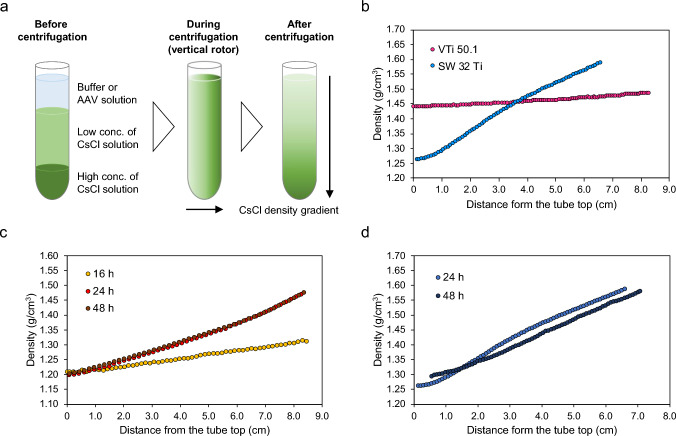
**a** Scheme of CsCl-DGUC purification from layering of solutions before centrifugation to the CsCl density gradient formation after centrifugation. Black arrows show the direction of the gradient (centrifugal force during centrifugation). The density gradients under the conditions of **b** 18,000 rpm, 16 °C, and initial CsCl concentration of 3.5 M (1.445 g/cm^3^) at 24 h using VTi 50.1 and SW 32 Ti, **c** 50,000 rpm, 16 °C, and initial CsCl concentration of 2.5 M at 16, 24, and 48 h using VTi 50.1, and **d** 18,000 rpm, 16 °C, and initial CsCl concentration of 3.5 M at 24 and 48 h using SW 32 Ti. All density values were obtained from refractive index measurements

In CsCl-DGUC for rAAV purification, various conditions were selected including rotor type, rotor speed, and initial CsCl concentration. The formed CsCl density gradient greatly differed depending on the chosen conditions. We examined the optimized condition for CsCl-DGUC for rAAV purification using a VTi 50.1 vertical rotor, initially testing the condition routinely used for a swinging-bucket rotor. In subsequent experiments, CsCl-DGUC was initiated from three distinct layers before centrifugation as shown in Fig. 3a. Here, it is worth noting that in a vertical rotor, the CsCl density gradient is formed in the tube perpendicular to the centrifugal force, and this gradient then transitions from the top to the bottom of the tube after centrifugation. Under this condition, the appropriate CsCl density gradient was not formed because the rotor speed was too low for a vertical rotor (Fig. 3b). We next tested the highest possible rotor speed and modified the initial CsCl concentration. The density gradient that formed at 24 h was found to be suitable for the separation of FPs and EPs as shown in Fig. 3c. The time required for the CsCl density gradient to reach equilibrium is theoretically described by the following Eq. 1.

1 $$t =k{({r}_{b}-{r}_{t})}^{2}$$

where *t* is the time in hours; *k* is a constant that depends on the diffusion coefficient and viscosity of the solute and the temperature; and *r*_*b*_ and *r*_*t*_ are the radius from the center of rotation to the bottom and top of the solution that forms a gradient, respectively. In DGE-AUC, the CsCl density gradient at 20 °C reaches equilibrium in approximately 12 h (Hirohata et al. 2024). The value of constant *k* is determined to be 9.9 from this information, and the time to reach equilibrium in both a vertical and a swinging-bucket rotor can be calculated based on a filled tube. The equilibrium times were 68 h and 756 h for the vertical and swinging-bucket rotors, respectively. Then, we experimentally determined the actual time to reach equilibrium using both rotors without AAV particles. As shown in Fig. 3c, the density gradient using a vertical rotor reached equilibrium at 24 h. The initial three-layer formation was considered to reduce the centrifuge run time to reach equilibrium from the calculated time since the starting point is closer to the expected final gradient. For the swinging-bucket rotor, equilibrium had not been reached in 48 h, as shown in Fig. 3d. The expected time from the calculation was also taken into account, the centrifugation run time to reach equilibrium was longer for a swinging-bucket rotor. In the case of vertical rotors, consequently, the CsCl-DGUC condition can be optimized for the separation of FPs and EPs under the CsCl density gradient that reaches equilibrium in the shorter centrifugation time.

**Fig. 4:**
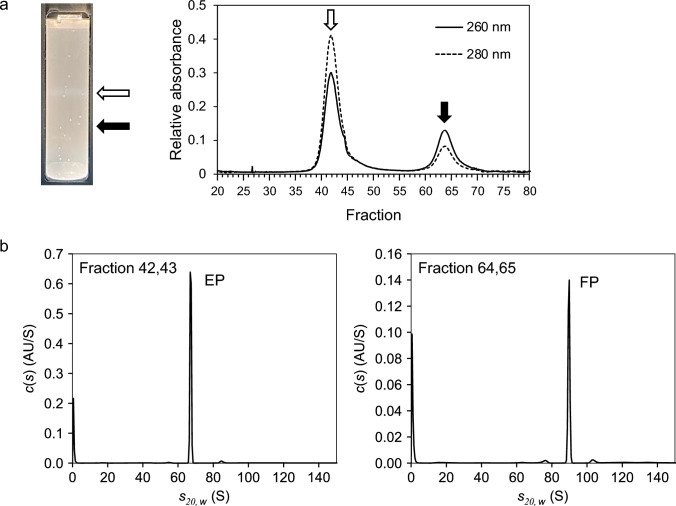
**a** CsCl-DGUC purification using VTi 50.1. White and black arrows show the bands corresponding to AAV2-EP and AAV2-CMV-EGFP, respectively. The absorbance profile at UV wavelength of 260 nm (solid line) and 280 nm (dashed line) during fractionation. **b** *c*(*s*) distributions of fractionated AAV2-EP (left) and AAV2-CMV-EGFP (right) obtained by SV-AUC at UV wavelength of 230 nm

Following the above examinations of CsCl-DGUC conditions, we performed CsCl-DGUC purification using a vertical rotor to evaluate FP/EP separation for the mixture of AAV2-CMV-EGFP and AAV2-EP. Clear separation of two bands regarded as EPs and FPs was observed based on A260/A280 (Fig. 4a), and we confirmed the purity of fractionated EPs and FPs after CsCl-DGUC by sedimentation velocity analytical ultracentrifugation (SV-AUC). As shown in Fig. 4b, highly purified FPs (and also EPs) were recovered in CsCl-DGUC using a vertical rotor. Although the volume of the AAV2 sample in this experiment was even small compared with laboratory-scale samples, much larger volumes of FPs and EPs must be separated from an rAAV manufacturing perspective. However, a formed shallow CsCl density gradient was best in this tube length for the separation between the FP and EP bands. From this viewpoint, vertical rotors have a great potential to recover highly purified FP from an unpurified rAAV sample containing large quantities of FPs and EPs. In addition, the number of tubes and the nominal capacity of VTi 50.1 in one run (12 $\times$ 39 mL $=$ 468 mL) are greater than those of a swinging-bucket rotor. These results indicate that VTi 50.1 can provide a higher throughput of CsCl-DGUC purification even for large-scale rAAV production.

### Examination of the influence of CsCl-DGUC purification process on the in vitro transduction efficiency of rAAV

**Fig. 5:**
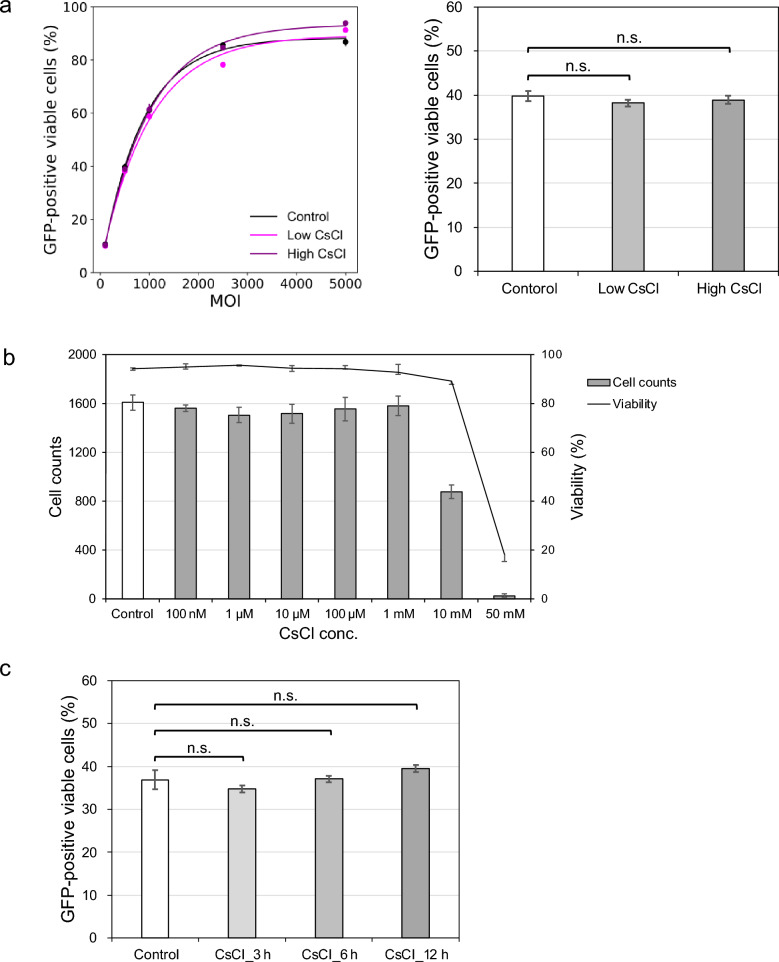
**a** Effect of residual CsCl on transduction efficiency of AAV2-CMV-EGFP in HeLaRC32 cells. The right graph shows the results at a MOI of 5 × 10^2^. **b** Dose-dependent relationship between cell viability and the supplemented CsCl concentration in HeLaRC32 cells. **c** Effect of exposure to a high CsCl concentration on transduction efficiency of AAV2-CMV-EGFP in HeLaRC32 cells. The graph shows the results at MOI 5 × 10^2^. Sample solution was exchanged to the original buffer by dialysis after exposure to 3 M (1.382 g/cm^3^) CsCl solution

To examine the influence of residual CsCl on the efficacy of rAAV, the transduction efficiency in HeLaRC32 cells was evaluated using AAV2-CMV-EGFP. For AAV2 samples supplemented with low and high concentrations of CsCl (quantified by inductivity coupled plasma mass spectrometry (ICP-MS), Table 1), the percentage of GFP-positive viable cells did not differ significantly from control AAV2, as shown in Fig. 5a. In addition, we assessed the viability of HeLaRC32 cells after exposure to CsCl. As shown in Fig. 5b, adding 1 M CsCl (final concentration in the medium: 10 mM) decreased cell viability, whereas a final concentration of less than 1 mM CsCl in the medium did not influence cell viability. Thus, we confirmed that the residual level of CsCl (i.e., < 1 mM) does not exert cytotoxicity, and these observations were consistent with a previous study (Kobayashi et al. 2017). Furthermore, AAV2 immersed in 3 M CsCl solution for 1, 3, or 7 days at 4 °C followed by a dialysis step. The amounts of residual CsCl were also determined by ICP-MS as shown in Table 1, which were equivalent to the concentration of control sample (9.90 ppb). For this reason, we demonstrated that CsCl was completely removed by the dialysis step after CsCl-DGUC.

**Table 1:** The amount of residual CsCl in AAV samples determined by ICP-MS measurement

AAV2 sample	CsCl conc. in the sample	CsCl conc. in HeLa cell (× 100 dilution)
Control	9.90 ppb (74.5 nM)	745 pM
Low CsCl	1.167 ppm (8.8 µM)	88 nM
High CsCl	1388 ppm (10.4 mM)	104 µM
3 M CsCl for 1 day*	12.95 ppb (97.4 mM)	–
3 M CsCl for 3 day*	6.45 ppb (48.5 mM)	–
3 M CsCl for 7 day*	5.35 ppb (40.3 mM)	–

*AAV samples were immersed in 3 M CsCl followed by a dialysis step

Finally, we examined the influence of exposure to a high CsCl concentration during CsCl-DGUC on rAAV. To mimic CsCl-DGUC, AAV2 samples were immersed in 3 M CsCl solution for 3, 6, and 12 h at 4 °C followed by a dialysis step. The percentage of GFP-positive viable cells did not show a significant difference (Fig. 5c). In summary, the expected level of CsCl exposure during CsCl-DGUC did not cause a significant decrease in transduction efficiency in vitro.

## Conclusions

CsCl-DGUC enables high-resolution separation of FPs and EPs compared with other techniques for rAAV purification. Here, we reported that the existence of encapsidated DNA in AAV capsid is the cause of the buoyant density differences of FPs with different VP stoichiometries. Moreover, we demonstrated that the degree of separation depends on the length of encapsidated DNA. Given that VP stoichiometry in FPs correlates with transduction efficiency, CsCl-DGUC can be used for the recovery of highly purified and highly active FPs. Traditionally, swinging-bucket or fixed-angle rotors have been employed for CsCl-DGUC. However, vertical rotors offer numerous benefits including improved purity and higher throughput that are required for viral vector manufacturing in gene therapy. We further demonstrate sufficient safety and efficacy of rAAV after CsCl-DGUC. The findings in this study advance the practical use of CsCl-DGUC in rAAV purification.

## Materials and methods

### rAAV production

HEK293F cells (Viral Production Cells 2.0, Thermo Fisher Scientific, Waltham, MA, USA) were used for rAAV production. HEK293F cells were maintained with BalanCD HEK293 (FUJIFILM Irvine Scientific, Inc., Santa Ana, CA, USA). HEK293F cells were grown as adherent cultures in 5% CO_2_ at 37 °C. All AAV samples were generated using triple-plasmid co-transfection. In brief, pAAV-Rep&Cap (serotypes 2 and 8), pAd helper, and transgene plasmid (CMV-EGFP) vectors were co-transfected into suspended HEK293F cells cultured in a flask at a ratio of 1:1:1. FectoVIR-AAV (Polyplus, Illkirch, France) was used as the transfection reagent. AAV samples from the transfected cells and the medium were harvested 96 h post-transfection and purified by affinity chromatography using AAVX columns (Thermo Fisher Scientific). Then, AAV samples were subjected to CsCl-DGUC using an Optima XE-90 and a SW 41 Ti rotor (Beckman Coulter, Brea, CA, USA) at 16 °C. FP and EP fractions were collected using a Piston Gradient Fractionator (BioComp Instruments, Fredericton, Canada) equipped with a Triax Flow Cell (BioComp Instruments) monitoring UV at 260 and 280 nm. Finally, fractions were dialyzed in PBS (Thermo Fisher Scientific), 200 mM NaCl (FUJIFILM Wako Pure Chemical, Osaka, Japan), and 0.001 w/v% poloxamer 188 (BASF, Ludwigshafen, Germany) using Slide-A-Lyzer MINI Dialysis Devices (Thermo Fisher Scientific).

### Molecular weight and partial-specific volume calculation for AAV8

The theoretical values of Mw and *vbar*_aq_ for AAV8 FPs and EPs were determined according to a previously published method (Hirohata et al. 2024). Briefly, the Mw of EP was calculated from the amino acid sequences of VP1, VP2, and VP3 for each VP stoichiometry, and *vbar*_aq_ values were determined from the sequences using the program SEDNTERP (Philo 2023). The Mw for FP was the sum of the Mw values for EP and ssDNA calculated from the nucleotide sequence. The *vbar*_aq_ for FP can be theoretically calculated using the following equation:

2 $${vbar}_{FP}=\frac{{vbar}_{EP}\times {Mw}_{EP}+{vbar}_{ssDNA}\times {Mw}_{ssDNA}}{{Mw}_{EP}+{Mw}_{ssDNA}}$$

where *vbar*_FP_, *vbar*_EP_, and *vbar*_ssDNA_ are the *vbar*_aq_ for FP, EP, and ssDNA, respectively. We used 0.52 cm^3^/g as *vbar*_ssDNA_ based on a previous study (Hirohata et al. 2024). Mw_FP_, Mw_EP_, and Mw_ssDNA_ are the Mw values for FP, EP, and ssDNA, respectively.

### AUC analysis

UV–vis measurements were performed using a BioMate 160 UV–Vis spectrophotometer (Thermo Fisher Scientific) and data were collected at 210–350 nm every 1 nm using a 1-cm light path cuvette. DGE- and SV-AUC experiments and analyses were performed as described in a previous study (Hirohata et al. 2024).

Briefly, for DGE-AUC, AAV8-CMV-EGFP and AAV8-EP were dissolved in PBS supplemented with 2.75 M (1.351 g/cm^3^) CsCl. A volume of 390 µL of AAV sample was loaded into a sample sector equipped with sapphire windows and a 12-mm double-sector charcoal-filled epon centerpiece (Beckman Coulter). In addition, a volume of 400 µL of corresponding CsCl/PBS buffer was loaded into a reference sector. Data were collected at 20 °C using an Optima AUC (Beckman Coulter) with a UV detection system, with the wavelength for UV detection set at 230 nm. Data were collected every hour with a radial increment of 10 µm.

For SV-AUC, AAV2-CMV-EGFP and AAV2-EP were diluted to a final absorbance at a 1-cm path length of approximately 0.2 and 0.8 at 230 nm, respectively. A volume of 390 µL of AAV sample or 400 µL of buffer was loaded into a sample sector or reference sector, respectively. The sapphire windows and a 12-mm double-sector charcoal-filled epon centerpiece were used. Data were collected at 20 °C using an Optima AUC at 10,000 rpm with a detection wavelength of 230 nm. Data were collected with a radial increment of 10 µm every 150 s. The sedimentation data were analyzed using the continuous *c*(*s*) distribution model of the program SEDFIT (version 16.2b) (Schuck 1998), where the frictional ratio, the meniscus, the time-invariant noise, and the radial-invariant noise were fitted using a regularization level of 0.68. A sedimentation coefficient range of 0–250 S was evaluated with a resolution of 500, and buffer density and viscosity were calculated using the program SEDNTERP. The apparent sedimentation coefficients were converted to the sedimentation coefficients in water at 20 °C (*s*_20,w_) using the buffer density and viscosity, and the partial-specific volumes of AAV2-CMV-EGFP and AAV2-EP. Figures of the *c*(*s*) distribution were generated using the program GUSSI (version 1.3.2) (Brautigam 2015).

### CsCl-DGUC

When CsCl-DGUC was performed using a swinging-bucket rotor, the total CsCl concentration was adjusted to 3.5 M using three-layer formation before centrifugation as follows. For the buffer preparation, OptiMATE CsCl (Beckman Coulter) was used. To an Open-Top Ultra-Clear Tube (Beckman Coulter), 12 mL of 5 M CsCl/PBS buffer was added, then 21 mL of 3.5 M CsCl/PBS buffer, followed by 5 mL of PBS was carefully layered on top, forming a clear interface. CsCl-DGUC was performed in a SW 32 Ti rotor (Beckman Coulter) at 18,000 rpm for 24 and 48 h at 16 °C. After centrifugation, the solution was continuously fractionated and collected as 0.4-mL fractions using a Piston Gradient Fractionator. The refractive indexes (RIs) of each fraction were measured using Abbemat 200 (Anton Paar, Graz, Austria) and converted to densities as described in the previous study (Hirohata et al. 2024).

When CsCl-DGUC was performed using a vertical rotor, the total CsCl concentration was adjusted to 2.5 M. To a Quick-Seal Centrifuge Tube (Beckman Coulter), 10.7 mL of PBS was added, then 19 mL of 2.6 M (1.332 g/cm^3^) CsCl/PBS buffer, followed by 9.7 mL of 5 M (1.633 g/cm^3^) CsCl/PBS buffer was loaded from the bottom of the tube, forming a clear interface. When the mixture of AAV2-CMV-EGFP and AAV2-EP was analyzed, the AAV solution was carefully applied between the PBS and 2.6 M CsCl/PBS layers. After the tube top had been sealed using a Tube Topper Kit (Beckman Coulter), CsCl-DGUC was performed in a VTi 50.1 rotor (Beckman Coulter) at 50,000 rpm for 16–48 h at 16 °C. The condition employed for SW 32 Ti was also applied for VTi 50.1. After centrifugation, the solution was continuously fractionated from the bottom of the tube using a Fraction Recovery System (Beckman Coulter) or the tube was cut using a Tube Slicer (Beckman Coulter) and fractions were collected automatically. The RIs of each fraction were measured and fractions containing FPs and EPs were dialyzed for the SV-AUC experiments.

### Genomic titer determination

rAAV samples were diluted in dilution buffer, which consisted of DNase I Buffer, DNase I (Takara Bio, Shiga, Japan), and 0.05 w/v% poloxamer 188. The mixtures were incubated at 37 °C for 30 min to digest DNA contaminants outside the AAV capsid. DNase I was inactivated by adding EDTA (pH 8.0; NIPPON GENE, Tokyo, Japan) to a final concentration of 50 mM at 25 °C for 5 min. The samples were then heated at 95 °C for 10 min to denature the AAV capsid. The pretreated samples were diluted with 0.001 w/v% poloxamer 188 in TE buffer (AM9849, Invitrogen, Waltham, MA, USA) to the required concentration. Digital polymerase chain reaction (dPCR) was performed as a duplex assay using specific forward and reverse primers (900 nM) and a specific probe (250 nM) that targeted the ITR using a fluorescein amidite fluorophore (Hokkaido System Science, Hokkaido, Japan). Reaction mixes comprised primers, probes, nuclease-free water, and QuantStudio Absolute Q DNA Master Mix (Thermo Fisher Scientific). dPCR was performed using a QuantStudio Absolute Q Digital PCR System and 16-well microfluidic array plates (Thermo Fisher Scientific). The dPCR consisted of enzyme activation at 94 °C for 10 min, followed by 40 cycles of 94 °C for 5 s and 54 °C for 30 s. The following primers and probes were used: ITR forward primer 5′-GGAACCCCTAGTGATGGAGTT-3′; ITR reverse primer 5′-CGGCCTCAGTGAGCGA-3′; and ITR probe 5′-[FAM]-CACTCCCTCTCTGCGCGCTCG [BHQ1]−3′.

### Flow cytometry

HeLaRC32 cells were used for the evaluation of in vitro transduction efficiency. HeLaRC32 cells were maintained in Dulbecco’s modified Eagle’s medium (Sigma-Aldrich, Burlington, MA, USA) supplemented with 10% fetal bovine serum (HYCLONE, Marlborough, MA, USA) and 1% penicillin–streptomycin (FUJIFILM Wako Pure Chemical), and grown as adherent cultures in 5% CO_2_ at 37 °C. To assess the cytotoxicity of residual CsCl, cells were cultured in the final concentration of 100 nM, 1 µM, 10 µM, 100 µM, 1 mM, 10 mM, and 50 mM CsCl in the medium, respectively (*n* = 3). The viable cells were counted using the Vi-CELL BLU (Beckman Coulter). HeLaRC32 cells were seeded at 5 × 10^4^ cells/well in 24-well culture plates. Cells were infected with rAAV at a multiplicity of infection (MOI) of 1 × 10^2^, 5 × 10^2^, 1 × 10^3^, 2.5 × 10^3^, and 5 × 10^3^ in triplicate. Cells were incubated at 37 °C for 48 h and then harvested. The percentage of viable cells expressing EGFP was determined using the CytoFLEX flow cytometry system (Beckman Coulter).

### ICP-MS

Standard solutions were prepared between 0 and 50 ppb by diluting the cesium standard solution (Fujifilm Wako Pure Chemical). The sample and the standard solutions were diluted with 5% nitric acid. The cesium concentration in samples was measured using ICP-MS (Agilent 7900, Agilent Technologies, Santa Clara, CA, USA). Data analysis was performed using ICP-MS MassHunter 5.2 software (Agilent Technologies).

## Supplementary Information

Below is the link to the electronic supplementary material.

Supplementary file1 (DOCX 126 KB)

Supplementary file2 (XLSX 35 KB)

## Data Availability

Data supporting the results of this study are available from the corresponding author upon request.
