# Supplementary material for: Use of cesium chloride density gradient ultracentrifugation for the purification and characterization of recombinant adeno-associated virus
Source: Eur Biophys J. 2025 May 19;54(6):415–25. doi: 10.1007/s00249-025-01751-1 (PMC12552358; doi:10.1007/s00249-025-01751-1)
Supplement: Supplementary file 1 — Supplementary file1 (DOCX 126 KB) [file 249_2025_1751_MOESM1_ESM.docx]

**Supplementary Information**

**Use of cesium chloride density gradient ultracentrifugation for the purification and characterization of recombinant adeno-associated virus**

Kiichi Hirohata^1^, Shinichiro Kino^1^, Takuya Yamane^1,2^, Karin Bandoh^1^, Takeshi Bamba^2,3^, Shawn M. Sternisha^4^, Tetsuo Torisu^1^, Mitsuko Fukuhara^5^, Yuki Yamaguchi^1^, and Susumu Uchiyama^1,5*^

1. Department of Biotechnology, Graduate School of Engineering, Osaka University, 2-1 Yamadaoka, Suita, Osaka, 565-0871, Japan

2. Institute of Metabolomics, BYU-Analytica Inc., Suita, Osaka, 565-0871, Japan

3. Division of Metabolomics/Mass Spectrometry Center, Medical Research Center for High Depth Omics, Medical Institute of Bioregulation, Kyushu University, Fukuoka, Fukuoka, 812-8582, Japan

4. Beckman Coulter Life Sciences, Indianapolis, IN, USA

5. U-Medico Inc., 2-1 Yamadaoka, Suita, Osaka 565-0871, Japan

* Corresponding author: Susumu Uchiyama, Ph.D. E-mail: suchi@bio.eng.osaka-u.ac.jp

**Fig. S1** DGE-AUC equilibrium profiles of (a) AAV8-CMV-EGFP and (b) AAV8-EP under the conditions of 2.75 M CsCl and 42,000 rpm. FP1 and FP2 are two populations of AAV8-CMV-EGFP with low and high buoyant densities, respectively. Both results were detected at a UV wavelength of 230 nm.
